# A mechanism-driven real-time respiratory modulation framework for rapid affective regulation via prefrontal EEG computational phenotyping

**DOI:** 10.3389/fpsyt.2026.1879147

**Published:** 2026-06-29

**Authors:** Minglei Sun, Haichuan Wu, Ming Deng, Qingxing Qu

**Affiliations:** 1 School of Industrial Design, Lu Xun Academy of Fine Arts, Shenyang, Liaoning, China; 2 School of Mechanical Engineering and Automation, Northeastern University, Shenyang, Liaoning, China; 3 School of Business Administration, Northeastern University, Shenyang, Liaoning, China

**Keywords:** affective regulation, computational phenotyping, electroencephalogram (EEG), psychomotor marker, respiratory modulation

## Abstract

Respiratory modulation has emerged as a measurable psychomotor phenotype and a promising non-invasive approach for the regulation of affective disturbances. In this study, a mechanismdriven real-time respiratory modulation framework is proposed to investigate its rapid effects on affective states and underlying neurophysiological dynamics in the context of computational psychiatry. A total of 26 breathwork-naive participants were recruited, of whom 24 were retained for final analysis after two EEG datasets with excessive noise were excluded; all participants completed a 40-minute intermittent real-time respiratory modulation protocol comprising two 20-minute sessions under controlled laboratory conditions. Multimodal assessments, including electroencephalography (EEG) and the Profile of Mood States (POMS), were employed to quantify computational phenotypes associated with changes in affective states before and after the intervention. EEG signals were analyzed with a focus on prefrontal oscillatory activity, examining band-specific power variations associated with affective processing. Behavioral results indicated significant improvements in affective states, characterized by reduced negative psychological symptoms (e.g., tension and fatigue) and enhanced positive mood (*p <* 0.05). EEG analysis revealed decreased FZ beta-band power and reduced FZ/FCZ beta-band Midline Differential Index (*p <* 0.05), while theta-band changes were non-significant, indicating rapid cortical EEG phenotypes of affective regulation. The findings suggest that short-duration controlled respiration may induce measurable and rapid modulation of both subjective states and neural computational phenotypes of emotion. This study provides preliminary evidence for a mechanism-driven link between psychomotor respiratory patterns and prefrontal EEG oscillations, and supports the development of real-time biofeedback systems and respiration-based interventions for psychiatric evaluation and adaptive affective regulation.

## 1 Introduction

Affective disturbances and their accompanying psychomotor abnormalities in psychiatric disorders represent core challenges in current clinical psychiatry (1). To achieve precise assessment and intervention for such disorders, exploring computational phenotyping based on objective physiological and behavioral signals has become a crucial research direction (2). Among various physical and physiological phenotypes, alongside macroscopic motor representations such as gait, respiration serves as a unique physiological motor rhythm capable of both direct voluntary control and reflex regulation, making it a key indicator of an individual’s affective state (3). Respiration not only functions in oxygen delivery but is also deeply intertwined with affective processing, therefore, conscious respiratory modulation is of significant importance to both physiological health and affective well-being (4, 5). Moreover, respiratory modulation training is simple, minimally invasive, and rarely constrained by cultural differences. Consequently, it has garnered considerable attention in clinical medicine and healthcare rehabilitation as a non-pharmacological intervention strategy (6, 7). Studies indicate that conscious respiratory modulation techniques can significantly impact physiological and affective states by influencing autonomic nervous system function, altering cortical activity, and improving blood circulation. Specifically, modulating respiratory frequency and depth can effectively lower blood pressure, regulate heart rate variability (8), alleviate anxiety, and enhance attention levels (9, 10).

A profound bidirectional relationship exists between respiration and human affective states. Early affective computing studies demonstrated that specific respiratory patterns could distinguish different emotional states, analyzing parameters such as respiratory rate and pause amplitude can objectively reflect the evolution of states like joy, sadness, and anxiety (11, 12). Variations in respiratory frequency and amplitude can induce significant transitions in affective states, shifting individuals from calm to excited or from relaxed to tense (13). Rapid and shallow breathing typically activates the sympathetic nervous system, thereby exacerbating anxiety and psychological tension (14, 15). Conversely, slow and deep breathing activates the parasympathetic nervous system, lowering heart rate and blood pressure to promote relaxation. This further confirms the direct regulatory role of respiration on affective experiences and states as a psychomotor signal (16, 17).

Systematic, long-term controlled breathing exercises, typically lasting 20–30 minutes daily for over eight weeks, yield significant psychological intervention effects on affective regulation, stress management, and cognitive function. Regarding affective regulation, long-term respiratory modulation training effectively improves mental states and reduces levels of anxiety, depression, and stress (18, 19). This enables individuals to better cope with negative emotions while elevating their overall affective state (20, 21). In contrast, real-time respiratory modulation refers to short-term (typically 15–20 minutes), targeted breathing adjustments (e.g., slow diaphragmatic or rhythmic breathing) performed during affective fluctuations or stressful situations. Its mechanism primarily relies on the rapid modulation of the prefrontal-amygdala neural circuit, thereby alleviating acute stress responses. Furthermore, breathing exercises play a vital role in the clinical treatment of patients with affective disorders and substance abuse (22, 23). In terms of stress management, deep slow-breathing exercises can effectively relieve stress and appropriately reduce the occurrence of negative psychological symptoms such as anxiety and depression, particularly in emergent conditions and high-pressure scenarios (24, 25).

Respiratory modulation techniques encompass various methods, commonly including slow deep breathing, yogic breathing, and mindful breathing, each yielding distinct effects on affective regulation (26, 27). Slow deep breathing is a frequently used technique characterized by a slow and deep respiratory frequency approaching 0.1 Hz. This method improves pulmonary gas exchange, increases oxygen intake, and concurrently triggers autonomic nervous system balance, thereby promoting physical relaxation and reducing perceived stress (28–30). Yogic breathing includes multiple practices, such as diaphragmatic breathing, bellows breath, and humming bee breath, each offering specific therapeutic benefits (31–33). Mindful breathing enhances awareness and attention by focusing on the respiratory process. It increases the activation of the prefrontal cortex, modifies resting-state functional connectivity in the amygdala, and mitigates individual stress responses. Consequently, it is widely utilized to manage pain, anxiety, and depression, exhibiting significant potential in improving health conditions, adaptive affective regulation capabilities, and overall well-being (34, 35).

Respiratory rhythms modulate affective processing through specific neurophysiological mechanisms (36, 37). Two key neural mechanisms are involved in regulating affect-related brain regions. First, slow breathing at a rate of 5–7 breaths per minute can synchronize Gamma (*γ*) band (30–100 Hz) neural oscillations between the prefrontal cortex and emotional centers, significantly enhancing the prefrontal cortex’s regulatory function over these regions (38, 39). Second, deep breathing activates chemosensory neurons in the nucleus tractus solitarius, which subsequently inhibits the overactivation of the noradrenergic system in the locus coeruleus. This effectively reduces sympathetic nervous system excitability, producing a rapid sedative effect. The brainstem respiratory centers share dense functional connectivity with affectregulating neural networks. These respiratory control centers not only integrate affective information from the amygdala to adjust respiratory patterns but also exert a reciprocal influence on the prefrontal-amygdala affective regulatory circuit through neural oscillatory coupling mechanisms (40, 41). The cerebral control of respiration exhibits a hierarchical architecture: the cortex is responsible for conscious respiratory modulation, while the pontomedullary network maintains autonomic rhythms. Moreover, respiratory rhythms can significantly influence the affective processing function of the prefrontal-amygdala circuit by modulating the synchronization of Gamma (*γ*) band neural oscillations (42).

Further investigations into specific neural mechanisms reveal that respiratory rhythms intricately affect the affective regulation process (43). For instance, the power of Alpha (*α*) oscillations is suppressed during inhalation and increased during exhalation within a respiratory cycle (*p <* 0.01). Deep breathing activates the parasympathetic nervous system, effectively inhibiting negative affective responses (44, 45). Additionally, olfactory sensory neurons directly modulate affective processing by activating the prefrontallimbic neural circuit (46, 47). For non-olfactory neurons, respiratory rhythms influence the firing patterns of neurons in the medial prefrontal cortex, thereby strengthening their inhibitory control over the amygdala (48, 49).

Neural signal detection technologies, such as electroencephalography (EEG) and functional magnetic resonance imaging (fMRI), continuously deepen our understanding of the intrinsic mechanisms of respiration. EEG studies related to respiratory modulation training indicate that the application of mindfulness and other breathing techniques increases Alpha (*α*) band power, reflecting an inward-focused, relaxed psychological state indicative of enhanced attention (50–52). Following coherent breathing training, the power of Delta (*δ*) and Theta (*θ*) bands significantly decreases, particularly in the parietal and temporal regions. Upon detailed frequency subdivision, the power of Beta1 (*β*_1_) and Beta2 (*β*_2_) oscillations in the parieto-temporal areas also exhibits a significant reduction. Conversely, Gamma (*γ*) band power increases post-training, and the alleviation of depressive symptoms is correlated with the reduction of Delta (*δ*) and Theta (*θ*) power in the occipital region (53, 54). Furthermore, multiple fMRI studies have demonstrated that controlled respiration can significantly alter blood-oxygen-level-dependent (BOLD) signals in key regions of the limbic system (55). Slow breathing (6 breaths/minute) enhances functional connectivity between the prefrontal cortex and the amygdala while simultaneously decreasing the activation intensity of the amygdala itself (56). Additionally, different respiratory patterns (e.g., diaphragmatic vs. thoracic breathing) induce specific activation patterns in the anterior cingulate cortex and insula, regions closely associated with affective regulation (57). These neuroimaging findings confirm the modulatory effects of respiratory training on affective neural circuits at a systemic level.

Functional near-infrared spectroscopy (fNIRS) research indicates that alternating deep and shallow respiratory patterns can significantly elevate oxygenated hemoglobin concentrations in the prefrontal cortex, this change is strongly correlated with improved affective regulation capabilities (58, 59). Leveraging its millisecond-level temporal resolution, magnetoencephalography (MEG) has confirmed that respiratory rhythms can modulate the phase synchronization of Gamma (*γ*) band (30–100 Hz) neural oscillations, particularly the functional connectivity of the prefrontal-amygdala circuit (51). Multimodal studies, such as concurrent EEG-fNIRS, further reveal that respiratory training enhances the coupling strength between prefrontal Theta (*θ*) oscillations (4–8 Hz) and hemodynamic responses, thereby boosting affective regulatory efficacy (60–62). Collectively, these findings provide multi-layered neuroimaging evidence for the mechanisms underlying respiratory interventions in affective regulation.

The aforementioned studies demonstrate that long-term respiratory modulation training (≥ 8 weeks, 20–30 minutes daily) can effectively improve affective states through slow-frequency breathing, diaphragmatic breathing, and mindfulness interventions (63). The underlying mechanisms include enhancing prefrontal regulatory functions and balancing the autonomic nervous system, which can significantly alleviate anxiety and depressive symptoms (18, 19). However, such protocols face notable limitations, including low compliance (30–40% of participants struggle to complete the full training cycle), slow onset of efficacy (affective regulation effects typically require 4–6 weeks to manifest), and insufficient standardization of parameters such as frequency, depth, and duration (22, 36). Consequently, there is an urgent need to develop more flexible, real-time modulation strategies. Current research highlights the significant potential of respiratory modulation as a non-pharmacological intervention in affective regulation, yet important challenges remain. Existing studies primarily focus on the cumulative effects of long-term training regimens (≥ 8 weeks) (18, 19), failing to adequately address the clinical demand for acute affective regulation. Research indicates that patients with anxiety disorders often find it difficult to complete systematic training during acute episodes (22), and the strict operational requirements of traditional methods limit their practical application (25). Furthermore, while neurophysiological studies reveal that different respiratory patterns exert varying regulatory effects on the prefrontal-amygdala circuit (36), existing protocols lack precise designs targeting acute affective disturbances.

Addressing the limitations and challenges of traditional respiratory training, this study proposes a mechanism-driven real-time respiratory modulation technique, with advantages spanning multiple critical dimensions. Regarding clinical applicability and innovation, the proposed real-time modulation optimizes respiratory parameters (6–10 breaths/minute, exhalation/inhalation ratio of 2:1 to 3:1) to achieve a rapid onset of effects, effectively countering the failure of long-term regimens in acute affective interventions. Utilizing personalized parameter settings based on CO_2_ tolerance tests (64), the intervention’s efficacy manifests through physiological indicators, such as a ≥ 25% increase in heart rate variability (HRV), within 3–5 minutes. This rapidly addresses the intervention needs for acute scenarios like anxiety attacks (22). In terms of neurophysiological precision, compared to the generalized effects of traditional protocols, the real-time modulation employs a specific “sighing” technique. This approach activates vagal pathways via the mechanical stimulation of diaphragmatic breathing to rapidly elevate prefrontal neural oscillatory power, thereby immediately shifting negative affective states. Furthermore, regarding operational procedural improvements, the proposed framework standardizes environmental controls (e.g., sound, light, temperature, and humidity) and simplifies the execution process to seated diaphragmatic breathing. This compresses the preparation and execution time from the lengthy sessions of traditional protocols to under 15–20 minutes per single session, successfully achieving the translational goal of a universally accessible and readily operable intervention.

This study specifically targets breathwork-naive individuals, defined as those without prior long-term training in voluntary respiratory affective regulation who may occasionally employ conscious breathing methods. To date, EEG technology, with its millisecond-level temporal resolution (1–5 ms), remains an irreplaceable research tool for directly reflecting neural mechanisms and revealing the spatiotemporal dynamics of respiratory modulation (51). Here, mechanism-driven denotes a respiratory protocol based on respiration–affect regulation mechanisms, including diaphragmatic breathing, prolonged exhalation, an exhalation/inhalation ratio of approximately 2:1 to 3:1, and CO_2_ tolerance based individualization. Real-time respiratory modulation refers to the two 20-minute laboratory-controlled respiratory sessions. Computational phenotypes are operationalized as POMS- and EEG-derived indices, including mood subscales, Total Mood Disturbance (TMD), prefrontal theta- and beta-band power, Frontal Asymmetry Index (AI), and Midline Differential Index (MDI), which were assessed using paired statistical comparisons. Therefore, this study employs experimental evaluations utilizing EEG measurements and affective state scales to objectively investigate the efficacy of real-time respiratory modulation. The findings offer valuable reference points for exploring affective regulation methodologies and developing related psychological counseling products. Notably, this research not only provides a novel perspective on non-pharmacological interventions for psychiatric affective disorders but also supplies critical empirical evidence and neurophysiological support for clinical treatments and digital therapeutic products aimed at psychological care. Ultimately, it contributes to the advancement of rapid intervention and management strategies for abnormal affective states within the mental health domain.

## 2 Methods

### 2.1 Participants

A total of 26 university students (17 males, 9 females, aged 19 to 24 years) from various institutions and academic disciplines were recruited for this real-time respiratory modulation experiment. Among the 26 recruited participants, two were excluded from the final analysis, resulting in a final analytic sample of 24 participants.The two excluded EEG datasets showed severe contamination from prominent ocular artifacts, which prevented reliable spectral feature extraction. All participants provided written informed consent prior to their inclusion in the study. A rigorous health screening protocol was implemented based on specific inclusion and exclusion criteria. Participants were required to be breathwork-naive, possessing no prior long-term experience in yoga or conscious respiratory training. Exclusion criteria included: a history of psychiatric or affective disorders (e.g., depression, anxiety, post-traumatic stress disorder), cardiovascular, cardiac, or respiratory diseases (e.g., hypertension, asthma), as well as other chronic illnesses, and any history of substance abuse or drug addiction. Furthermore, participants were instructed to completely abstain from alcohol, psychotropic medications, and caffeinated food or beverages for 24 hours preceding the experiment. Before the experiment, participants were asked to confirm by self-report that they had complied with these pre-experimental restrictions.

### 2.2 Data acquisition equipment

Neurophysiological assessments for evaluating the immediate effects of respiratory modulation were conducted using the Neuroscan electroencephalography (EEG) data acquisition system. Resting-state EEG signals were recorded via a Neuroscan Grael 45-channel DC amplifier (comprising 32 monopolar, 10 bipolar, and 3 auxiliary channels) paired with a Waveguard 32-channel EEG cap. Event triggers were automatically synchronized using the E-Prime 3.0 experimental platform, and data were continuously acquired via the CURRY 9 software at a sampling rate of 2048 Hz.

### 2.3 Affective assessment scale

Participants’ subjective affective states were evaluated using the Profile of Mood States (POMS) questionnaire. The POMS is a widely validated psychometric tool for self-evaluating psychological states, utilizing a 5-point Likert scale. While the original inventory comprises 60 descriptive items encompassing various sensory and emotional dimensions, this study employed a modified 40-item version to provide an objective estimation of the participants’ affective states. This refined version specifically targets five negative affective dimensions and two positive affective dimensions. Subsequent offline EEG data processing and neurophysiological analysis were performed utilizing the open-source EEGLAB toolbox within the MATLAB 2021b environment (65).

### 2.4 Respiratory modulation protocol

#### 2.4.1 CO_2_ tolerance testing for parameter personalization

To ensure participant safety and optimal experimental efficacy, the duration of the respiratory cycles was personalized based on individual lung capacity. This was achieved using a standard CO_2_ tolerance test (66). While seated in a relaxed posture, participants were instructed by the experimenter to complete four normal breathing cycles (one cycle defined as a full inhalation followed by a complete exhalation), ideally through the nose. Subsequently, participants took a maximal deep inhalation, once the lungs were fully expanded, they exhaled as slowly as possible through the nose or mouth. The total exhalation time (in seconds) required to empty the lungs was synchronously timed, with strict instructions not to hold their breath before the lungs were completely emptied. This recorded CO_2_ tolerance duration determined the individualized single breathing cycle level, as categorized in **Table 1**. In this study, the CO_2_ tolerance test was used only to determine individualized breathing cycle parameters for the subsequent respiratory modulation protocol, rather than as a primary outcome measure.

**Table 1:** Classification of CO_2_ tolerance time and breathing cycle duration.

Grade	CO_2_ tolerance time	Single breathing cycle duration level (s)
Level 1	0–20 s	3–4 s
Level 2	25–45 s	5–6 s
Level 3	50–70 s	8–10 s

#### 2.4.2 Respiratory technique

The real-time respiratory modulation technique was adapted from established slow deep breathing practices (64, 67). Participants maintained a natural, relaxed seated posture. They were instructed to perform diaphragmatic breathing, which relies on the active expansion of the abdominal cavity. During inhalation, the vertical diameter of the thoracic cavity increases, pushing the abdomen outward, during exhalation, the abdomen contracts to expel the air. To minimize sensory interference, the experimental environment was rigorously controlled, featuring sound attenuation, soft lighting, a comfortable room temperature, and neutral odors. Participants were guided to continuously reduce subjective conscious mental activity, clear their thoughts, and focus exclusively on their breathing rhythm and physical sensations.

The specific respiratory modulation procedure prioritized a sighing method, where the exhalation duration was deliberately longer than the inhalation duration. Specifically, the protocol emphasized prolonged exhalation, with the exhalation/inhalation duration ratio maintained at approximately 2:1 to 3:1, and no intentional breath-holding was allowed between inhalation and exhalation. If the inhalation phase lasted *a* seconds, the exhalation phase was prescribed to last 2*a* to 3*a* seconds. Based on the CO_2_ tolerance test results, the baseline parameter *a* was established to dictate the single cycle duration and the overall respiratory rate (typically 7 to 10 breaths per minute). For instance, an inhalation of 2 seconds would be paired with an exhalation of 4 to 6 seconds. Participants underwent a brief practice session to stabilize this specific respiratory pattern prior to the formal neurophysiological recording.

### 2.5 Experimental design

**Figure 1:**
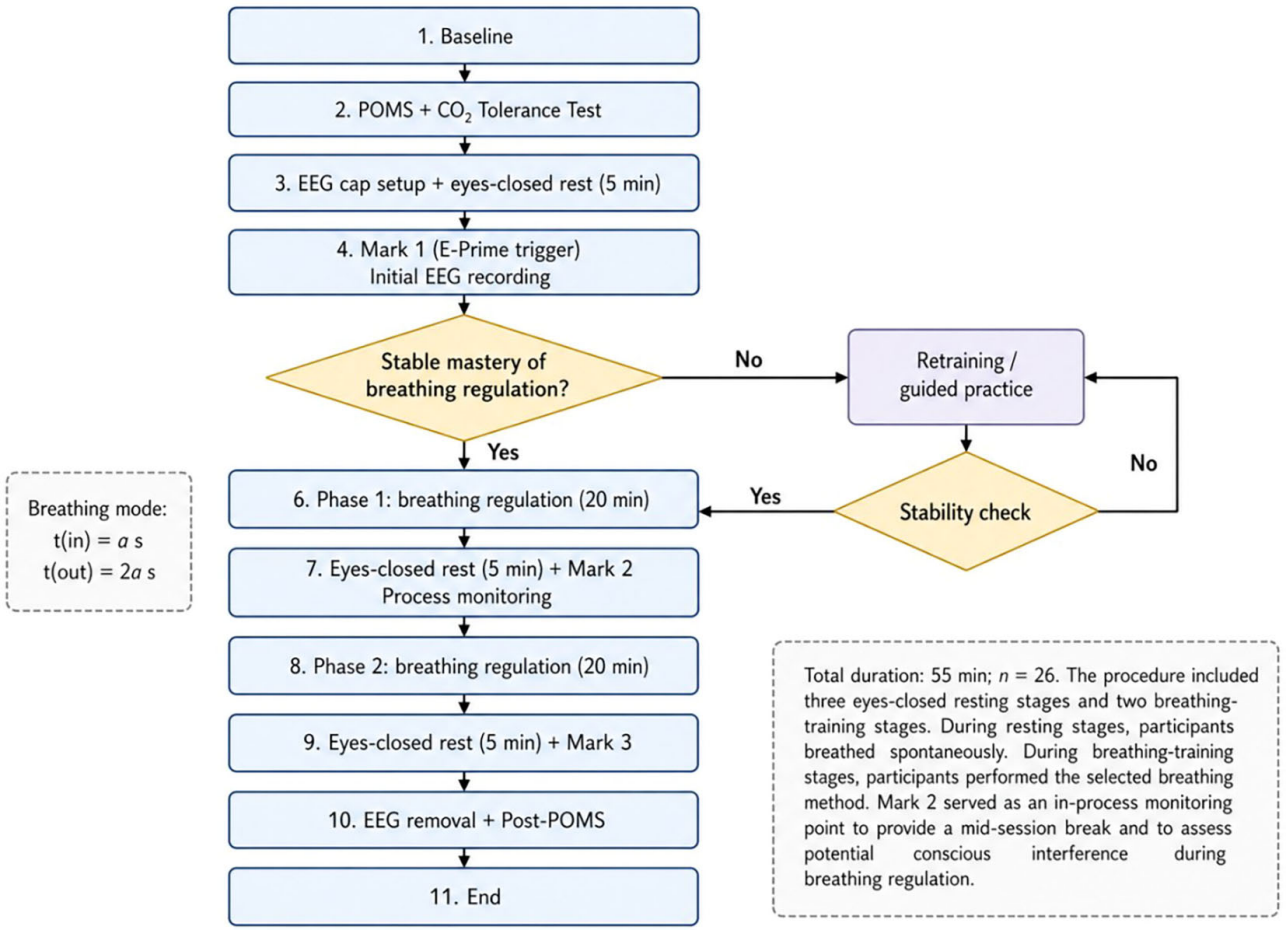
Experimental procedure of real-time respiratory modulation.

The intervention was standardized as a 40-minute intermittent real-time respiratory modulation protocol consisting of two 20-minute sessions. The real-time respiratory modulation experiment was conducted in three progressive stages. In the first stage, participants completed the POMS questionnaire and the CO_2_ tolerance test. After fitting the EEG cap, a 5-minute resting-state baseline was recorded (designated as *mark1* in E-Prime) to capture initial neurophysiological phenotypes and affective assessments. During all 5-minute resting-state EEG recordings, including *mark1*, *mark2*, and *mark3*, participants were instructed to keep their eyes closed, remain awake, sit still, relax their body, and avoid intentional movements or deliberate mental tasks. During the two 20-minute respiratory modulation sessions, participants kept their eyes open and followed the respiratory instructions while maintaining a relaxed seated posture. In the second stage, following familiarization with the respiratory modulation technique, participants engaged in the first 20-minute continuous respiratory modulation session. This was followed by a 5-minute resting-state recording (*mark2*). Participants then completed a second 20-minute respiratory modulation session, followed by a final 5-minute resting-state recording (*mark3*). In the third stage, EEG recording was terminated, the cap was removed, and participants completed a post-intervention POMS questionnaire to self-evaluate their immediate affective states (**Figure 1**). The 20-minute session duration was selected to align with typical human attention spans, as cognitive fatigue can influence respiratory modulation outcomes. The intermediate recording (*mark2*) served as a procedural monitor to assess the stability of the respiratory modulation procedure and to detect potential conscious interference, as illustrated in **Figure 1**.

### 2.6 Data analysis

#### 2.6.1 EEG preprocessing

Raw EEG signals are highly susceptible to ocular artifacts and external environmental interference, therefore, rigorous preprocessing is essential. In this study, EEG data preprocessing was performed using the EEGLAB toolbox within the MATLAB 2021b environment. After removing trigger channels and re-referencing the data to the common average reference, the EEG signals were downsampled from the original rate of 2048 Hz to 500 Hz to reduce computational complexity. A band-pass filter (0.5–51 Hz) was applied to retain the fundamental frequency components, and a notch filter (49–51 Hz) was utilized to suppress power-line noise. Bad channels were identified by visual inspection according to abnormal amplitude fluctuations, persistent signal drift, flat or nearly flat signals, excessive high-frequency noise, and poor electrode contact. These bad channels were removed before re-referencing to reduce potential bias. The cleaned continuous data were then re-referenced to a common average reference. Independent Component Analysis (ICA) was then conducted to eliminate ocular artifacts and other noise components. ICA components were rejected when their scalp topographies and time courses were consistent with stereotypical ocular artifacts, such as eye blinks or horizontal eye movements. Following artifact correction, 300-second continuous EEG epochs following *mark1* and *mark3* were extracted as pre- and post-intervention resting-state segments. The preprocessed EEG signals were then used for Fast Fourier Transform (FFT)-based frequency-domain feature extraction.

#### 2.6.2 EEG feature extraction

The preprocessed EEG signals were segmented by extracting 300-second continuous data epochs following *mark1* (pre-intervention baseline) and *mark3* (post-intervention). These epochs were utilized to calculate the mean Power Spectral Density (PSD, *µ*V^2^*/*Hz) for each channel, serving as the core computational phenotypes before and after the real-time respiratory modulation protocol.

This study focused on the Theta (*θ*) and Beta (*β*) bands as the primary frequency ranges for spectral analysis. The *θ* band is generally associated with attentional control, cognitive regulation, and affective processing, specifically, frontal midline *θ* activity reflects neural engagement during affective regulation and internal focus. Conversely, the *β* band correlates with wakefulness, alertness, and cognitive load, serving as an indicator of affective arousal and psychological tension. Previous neurophysiological studies on affective states have demonstrated that *θ* and *β* power act as crucial EEG phenotypes for differentiating affective states. Accordingly, PSD values for these bands were initially extracted for all 26 participants. Due to significant noise interference, the data of two participants were excluded, resulting in a final valid dataset of 24 participants.

Regarding electrode selection, four prefrontal and fronto-central channels (F3, F4, FZ, and FCZ) were chosen for targeted analysis. Channels F3 and F4, located in the left and right frontal regions respectively, are commonly employed for frontal asymmetry analysis to reflect lateralization during affective processing. Channels FZ and FCZ, situated in the frontal and fronto-central midline, are closely linked to attentional control and cognitive regulation. Previous research confirmed the methodological validity of these sites in affective EEG studies.

To evaluate relative power shifts between different frontal regions, two specific differential indices were computed based on the single-channel PSDs. The Frontal Asymmetry Index (AI) and Midline Differential Index (MDI) were calculated according to Equations 1, 2, respectively. First, to assess lateralized prefrontal activity, the Frontal Asymmetry Index (AI) was calculated. Following established methodologies for affective EEG lateralization (68), AI was defined as the difference between the natural logarithms of the mean PSDs of the left and right hemispheres:

(1) AI=ln (F3)−ln (F4)

Here, *F*3 and *F*4 represent the mean PSD of the corresponding frequency band at the respective electrodes. An *AI >* 0 indicates greater relative power in the left prefrontal region, whereas an *AI <* 0 denotes higher power in the right prefrontal region. As a dimensionless metric, comparing pre- and post-intervention AI values elucidates whether real-time respiratory modulation induces lateralized shifts in prefrontal activity.

Second, to observe relative power variations along the anteroposterior midline axis, the Midline Differential Index (MDI) was calculated for channels FZ and FCZ:

(2) MDI=ln (FZ)−ln (FCZ)

In this equation, *FZ* and *FCZ* denote the mean PSD at their respective locations. This metric quantifies the relative spatial distribution of neural oscillations between the frontal midline and the fronto-central regions. It serves as a supplementary index to evaluate spatial shifts in midline *β* or *θ* activity following the respiratory intervention. Similar to AI, MDI is a dimensionless logarithmic ratio.

#### 2.6.3 POMS data processing

Subjective affective data acquired from the POMS questionnaire before and after the real-time respiratory modulation were processed using SPSS software. The POMS items are scored on a 5-point Likert scale: 0 (Not at all), 1 (A little), 2 (Moderately), 3 (Quite a bit), and 4 (Extremely). The Total Mood Disturbance (TMD) score was calculated according to Equation 3:

(3) TMD=∑( Tension + Anger + Fatigue + Depression + Confusion )−∑( Vigor + Esteem )

Subsequently, paired-sample statistical analyses were conducted on the averaged scores of the 24 retained participants to assess affective shifts induced by the intervention.

#### 2.6.4 Statistical analysis

A within-subject experimental design was employed to compare EEG computational phenotypes and POMS scores before and after the respiratory modulation protocol. All statistical analyses were conducted on paired data (post-intervention minus pre-intervention baseline). Initially, the Shapiro-Wilk test was utilized to assess the normality of the differences. For normally distributed data, paired-sample *t*-tests were applied to determine statistical significance. Conversely, the Wilcoxon signed-rank test was utilized for non-parametric comparisons when the assumption of normality was violated. Specifically, comparisons were made for the mean PSD of *θ* and *β* bands at F3, F4, FZ, and FCZ, alongside the AI and MDI indices. Results are reported as mean ± standard deviation, with corresponding *t*-values or *Z*-values, and *p*-values. The threshold for statistical significance was defined *a priori* at *p <* 0.05. These procedures systematically evaluated the rapid affective regulation effects of real-time respiratory modulation on prefrontal neurophysiological dynamics and subjective affective states.

## 3 Results

### 3.1 Single-channel mean power spectral density analysis

The mean Power Spectral Density (PSD) of the Beta (*β*) and Theta (*θ*) bands at the FZ, FCZ, F3, and F4 channels were compared before and after the real-time respiratory modulation protocol. Within the *β* band, results indicated that only the FZ channel exhibited a statistically significant difference following the intervention. Specifically, the *β*-band power at FZ decreased from a pre-intervention baseline of 0.257 ± 0.142 to a post-intervention level of 0.221 ± 0.109. In contrast, the pre- and post-intervention differences for the *β* band at the FCZ, F3, and F4 channels did not reach statistical significance.

Regarding the *θ* band, no significant differences were observed across any of the four channels (FZ, FCZ, F3, and F4) following the intervention. Although the mean *θ* power at FZ and FCZ exhibited a slight post-intervention decrease, and the mean *θ* power at F3 and F4 showed a marginal increase, none of these fluctuations achieved statistical significance. These findings indicate that the immediate effects of real-time respiratory modulation on *θ*-band activity are highly variable, whereas its primary regulatory impact on EEG computational phenotypes is manifested through the suppression of *β*-band activity at the FZ channel, as detailed in **Table 2**.

**Table 2:** Comparison of Beta- and Theta-band mean PSD at FZ, FCZ, F3, and F4 before and after respiratory modulation (*N* = 24).

Channel	β Band mean ± SD		θ Band mean ± SD		β Statistics		θ Statistics	
	Pre	Post	Pre	Post	t/Z	p	t/Z	p
FZ	0.257 ± 0.142	0.221 ± 0.109	1.823 ± 1.336	1.596 ± 0.915	-2.114*^Z^*	0.034	-0.029*^t^*	0.997
FCZ	0.216 ± 0.102	0.222 ± 0.100	1.684 ± 1.160	1.614 ± 0.909	-0.736*^t^*	0.469	-0.286*^t^*	0.775
F3	0.287 ± 0.191	0.304 ± 0.183	1.075 ± 0.675	1.111 ± 0.661	-0.486*^t^*	0.632	-0.822*^t^*	0.420
F4	0.285 ± 0.253	0.276 ± 0.170	1.084 ± 0.753	1.141 ± 0.732	-0.629*^t^*	0.530	1.030*^t^*	0.314

Z denotes Wilcoxon signed-rank test values, t denotes paired-sample t-test values.

**Figure 2:**
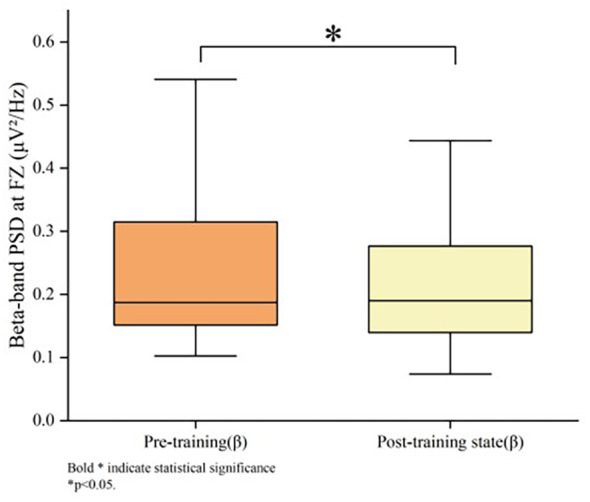
Pre- and Post-Intervention Comparison of Beta-Band Power at FZ (*N* = 24). * indicates statistical significance at p < 0.05.

Because the paired differences for the *β* and *θ* bands at the FZ channel violated the assumption of normality, the Wilcoxon signed-rank test was utilized. The results confirmed a significant reduction in *β*-band power at the FZ channel following the intervention (*Z* = −2.114, *p* = 0.034, effect size *r* = 0.43). This indicates that real-time respiratory modulation exerts a substantial effect on frontal midline *β* activity. Coupled with the descriptive statistics, this post-intervention reduction suggests that the respiratory protocol effectively mitigates neural activity associated with psychological tension, vigilance, and hyperarousal in the frontal midline region. Consequently, the primary neurophysiological response to real-time respiratory modulation in this context is characterized by alterations in *β*-band dynamics at the FZ channel (**Figure 2**).

### 3.2 Analysis of differential indices for beta and theta bands

To investigate lateralized shifts in prefrontal activity induced by the respiratory protocol, the Frontal Asymmetry Index (AI) was calculated and analyzed using paired-sample *t*-tests for both the *β* and *θ* bands. The results revealed no significant difference in the *β*-band AI before and after the intervention (*t*(23) = −0.596, *p* = 0.557, Cohen’s *d* = −0.122). Similarly, the *θ*-band AI exhibited no significant alteration (*t*(23) = 0.516, *p* = 0.611, Cohen’s *d* = 0.105). These findings indicate that real-time respiratory modulation does not consistently provoke lateralized asymmetric shifts in either *β* or *θ* prefrontal activity (**Table 3**).

**Table 3:** Comparison of Frontal Asymmetry Index (AI) and Midline Differential Index (MDI) before and after respiratory modulation (*N* = 24).

Index	Band	Pre mean (SD)	Post mean (SD)	t/Z	p
F3/F4 AI	*β*	0.057(0.523)	0.094(0.523)	−0.596t	0.557
	*θ*	0.003(0.264)	−0.009(0.306)	0.516t	0.611
FZ/FCZ MDI	*β*	0.147(0.385)	−0.011(0.217)	−2.771Z	0.006
	*θ*	0.065(0.564)	0.006(0.337)	−0.143Z	0.886

*Z* denotes Wilcoxon signed-rank test values, *t* denotes *t*-test values.

**Figure 3:**
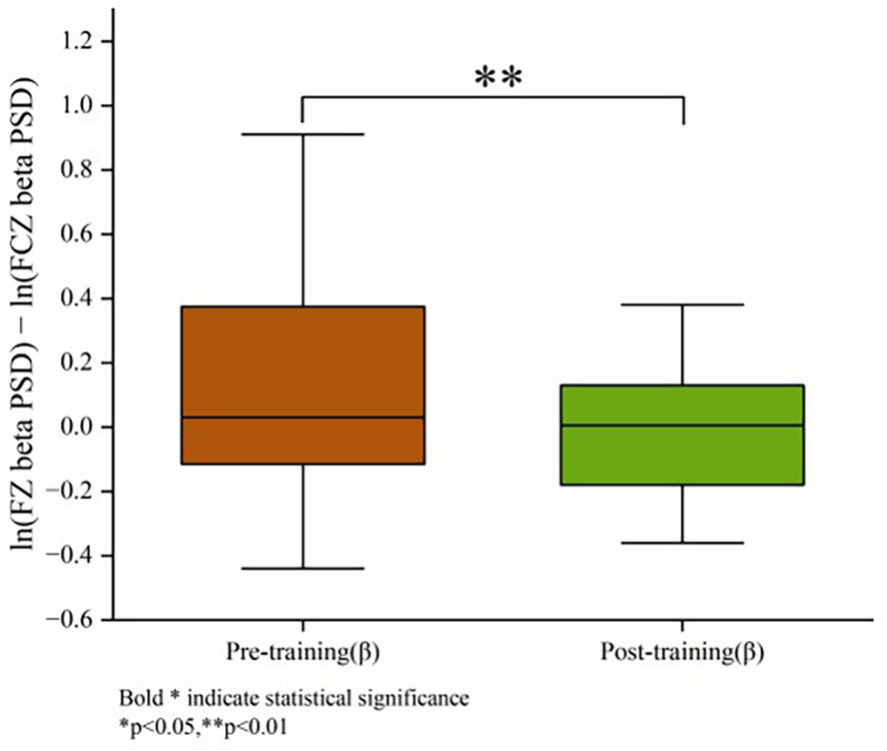
Pre- and post-intervention comparison of FZ/FCZ beta-band MDI (*N* = 24). ** indicates statistical significance at p < 0.01.

Furthermore, to examine the relative spatial shifts in *β* and *θ* oscillatory activity between the frontal midline and fronto-central regions, the MDI was analyzed using the Wilcoxon signed-rank test. A highly significant difference was identified in the *β*-band MDI following the respiratory intervention (*Z* = −2.771, *p* = 0.006, effect size *r* = 0.57). Rank analysis demonstrated that 17 out of the 24 participants exhibited a decreased *β*-band MDI post-intervention, indicating a robust downward trend in the relative power of FZ compared to FCZ. Conversely, the *θ*-band MDI showed no significant difference (*Z* = −0.143, *p* = 0.886), confirming that the intervention did not reliably alter the spatial distribution of *θ* power along the midline axis (**Figure 3**).

### 3.3 Qualitative analysis of EEG topographic maps

Qualitative assessment of the pre- and post-intervention EEG topographic maps reveals that the overall spatial distribution of the Beta (*β*) band remained largely consistent, characterized by relatively higher power in the posterior regions and lower power in the fronto-central regions. Following the intervention, however, the *β* band exhibited a modulatory trend in the fronto-central and left-central areas. Corroborated by the channel-level statistical results, which demonstrated a significant reduction at the FZ channel, this suggests that the respiratory modulation protocol exerts a regulatory effect on neural activity in these regions. These specific cortical zones are putatively associated with attentional control, somatic awareness, and sensorimotor regulation.

**Figure 4:**
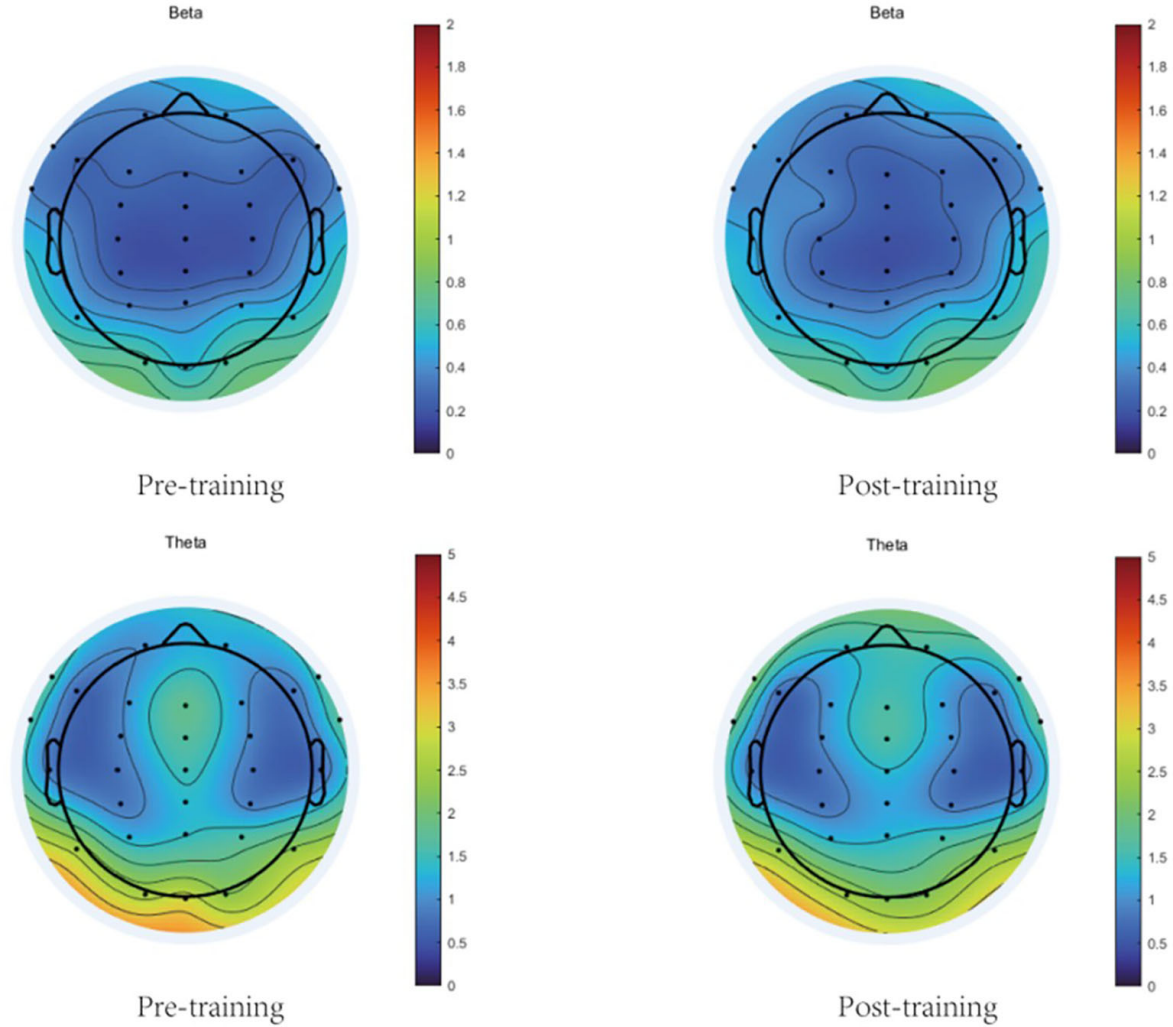
Pre- and post-intervention EEG topographic maps of beta and theta bands (*N* = 24).

Similarly, the Theta (*θ*) band consistently displayed a pronounced posterior distribution both before and after the intervention. Post-intervention, the *θ* band showed a descriptive, non-significant spatial tendency toward relative enhancement in the prefrontal area and a corresponding reduction in the occipital region. Therefore, this pattern should be interpreted cautiously as a trend-level observation rather than a definitive *θ*-band effect. As reflected in the topographic results, this *θ*-band alteration primarily manifests as a prefrontal power increase and an occipital decrease. However, because these variations did not achieve statistical significance, they should be interpreted cautiously as emergent trends (**Figure 4**).

The difference topographic maps further elucidate the relative spatial shifts in the *β* and *θ* bands induced by the respiratory intervention. The *β*-band difference map confirms observable alterations in the frontal midline, as well as the left fronto-central and central regions, with the FZ area specifically displaying a downward trend. Aligning with the significant statistical differences observed at the FZ channel, this indicates that the respiratory intervention substantively impacts *β* activity in fronto-central and left-lateralized sensorimotor areas.

**Figure 5:**
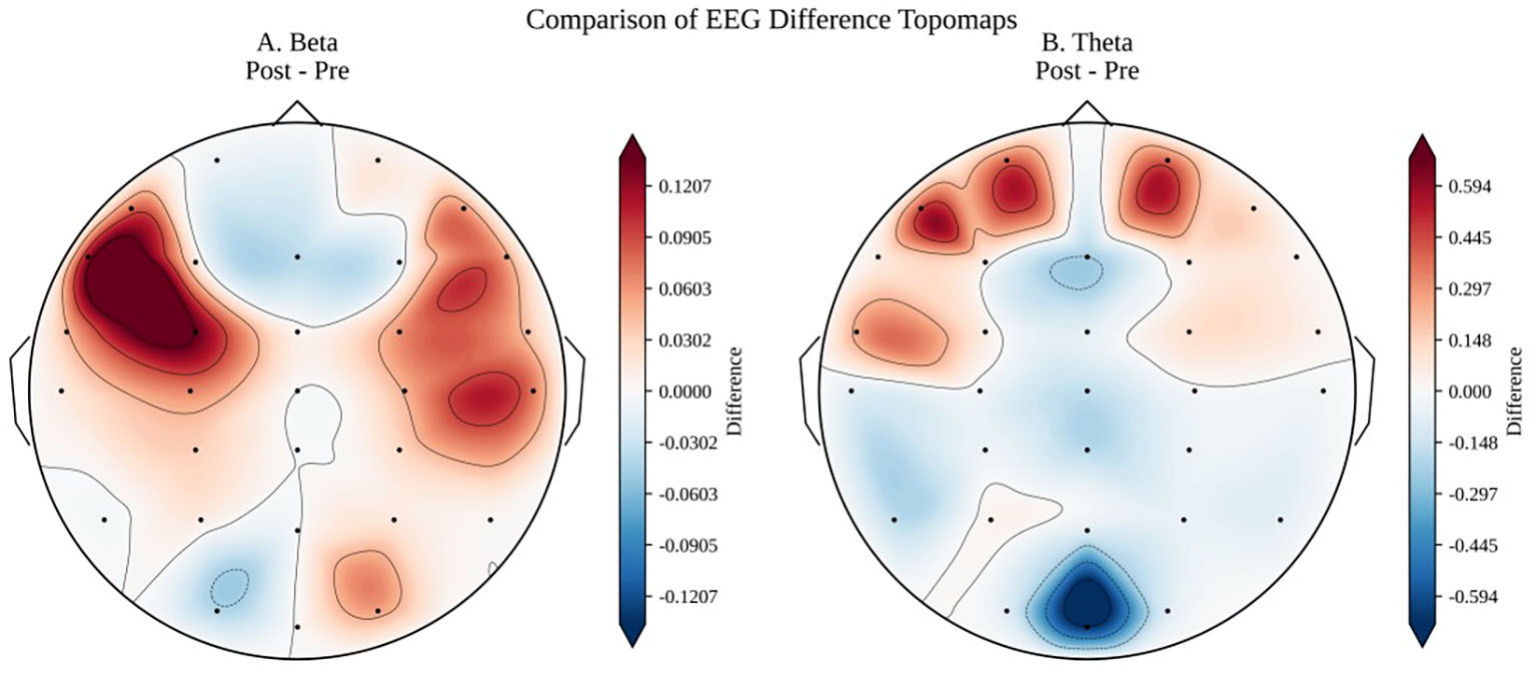
Post–Pre EEG difference topographic maps of **(A)** beta-band and **(B)** theta-band activity (*N = 24*).

Furthermore, the *θ*-band difference map highlights an upward trend in prefrontal *θ* activity and a downward trend in the occipital region, particularly near the OZ channel. This points to a potential spatial reconfiguration of *θ* oscillations. Nevertheless, consistent with the channel-level statistics, these *θ*-band differences did not reach the threshold for significance and are thus best discussed as spatial modulatory trends rather than definitive effects (**Figure 5**).

### 3.4 Analysis of subjective affective states (POMS)

Descriptive statistical analysis was conducted on the Profile of Mood States (POMS) scores of the 24 participants before and after the real-time respiratory modulation protocol. The results indicated a comprehensive reduction in multiple negative affective dimensions following the intervention. Specifically, the mean score for Tension decreased from 9.21 ± 5.36 to 3.38 ± 2.72, Anger decreased from 6.33 ± 6.95 to 2.13 ± 2.53, Fatigue dropped from 7.87 ± 5.30 to 4.50 ± 3.62, Depression was reduced from 7.54 ± 4.74 to 3.67 ± 2.16, and Confusion fell from 7.71 ± 4.23 to 3.71 ± 3.01. Consequently, the Total Mood Disturbance (TMD) score exhibited a substantial decline from a pre-intervention baseline of 18.92 ± 25.86 to a post-intervention level of −8.21 ± 12.53.

Concurrently, within the positive affective dimensions, participants demonstrated improvements in both Vigor and Self-esteem. The mean score for Vigor increased from 13.54 ± 6.06 to 15.00 ± 5.46, while Self-esteem rose significantly from 6.21 ± 1.69 to 10.58 ± 2.28. Overall, the subjective assessments confirm that the respiratory intervention effectively alleviated negative psychological symptoms and elevated positive affective states (**Table 4**).

**Table 4:** Descriptive statistics of POMS scores before and after respiratory modulation (*N* = 24).

Affective dimension	Pre-Intervention		Post-Intervention	
	Mean	SD	Mean	SD
Tension	9.21	5.36	3.38	2.72
Anger	6.33	6.95	2.13	2.53
Fatigue	7.87	5.30	4.50	3.62
Depression	7.54	4.74	3.67	2.16
Confusion	7.71	4.23	3.71	3.01
Vigor	13.54	6.06	15.00	5.46
Self-esteem	6.21	1.69	10.58	2.28
Total Mood Disturbance (TMD)	18.92	25.86	-8.21	12.53

To rigorously evaluate these affective shifts, paired-sample statistical tests were applied. The pairedsample *t*-test revealed that the Confusion score was significantly lower post-intervention (mean difference = −4.00 ± 3.92, *t*(23) = −4.995, *p <* 0.001). Although Vigor increased (mean difference = 1.46 ± 4.77), this improvement did not achieve statistical significance (*t*(23) = 1.497, *p* = 0.148).

**Figure 6:**
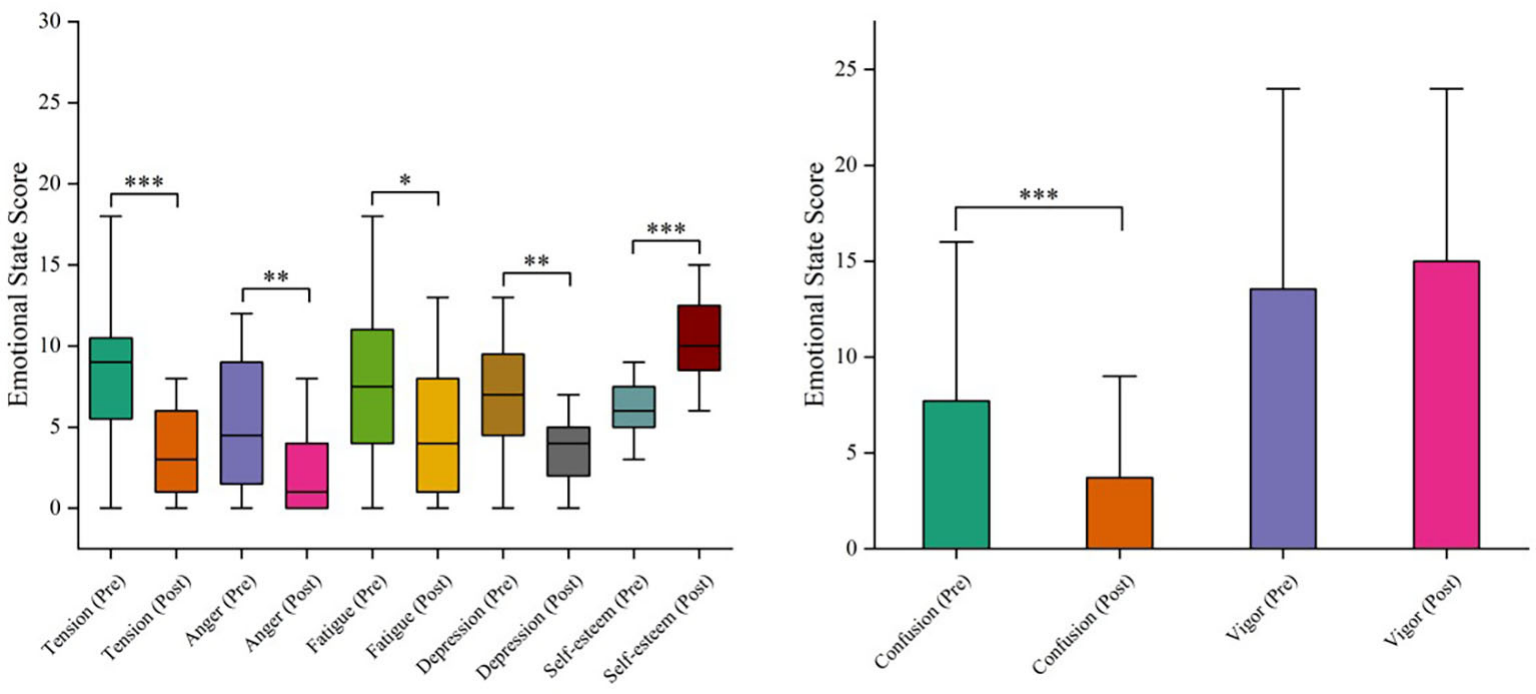
Pre- and post-intervention comparison of POMS affective state scores (*N* = 24). *p < 0.05, ** p < 0.01, *** p < 0.001.

For the remaining affective dimensions, the Wilcoxon signed-rank test was utilized to analyze the preand post-intervention differences. The results confirmed that following the respiratory modulation protocol, highly significant changes occurred across Tension, Anger, Fatigue, Depression, Self-esteem, and the Total Mood Disturbance (TMD) score. Specifically, significant reductions were observed in Tension (*Z* = −4.086, *p <* 0.001), Anger (*Z* = −3.467, *p* = 0.001), Fatigue (*Z* = −2.910, *p* = 0.004), and Depression (*Z* = −3.817, *p <* 0.001). Furthermore, Self-esteem was significantly enhanced (*Z* = −4.303, *p <* 0.001), culminating in a profound and significant decrease in the overarching TMD score (*Z* = −4.287, *p <* 0.001), as illustrated in **Figure 6**.

## 4 Discussion

This study investigated the rapid effects of a mechanism-driven, real-time respiratory modulation framework on subjective affective states and neurophysiological dynamics, utilizing psychometric questionnaires alongside EEG topographic mapping. The results demonstrate that following the short-term intervention, participants’ negative affective levels decreased significantly while their self-esteem notably improved. Concurrently, EEG analyses revealed specific spatial modulations in the *β* and *θ* frequency bands. Notably, *β*-band activity exhibited a downward trend primarily in the FZ region, while *θ*-band activity demonstrated a spatial reorganization characterized by prefrontal enhancement and occipital reduction.

### 4.1 Frontal midline beta suppression

Neurophysiological findings indicate a significant reduction in *β*-band power at the FZ channel postintervention, directly reflecting the attenuation of anxiety-related affective arousal within the frontal midline. Located in the frontal midline, the FZ region is intrinsically associated with attentional control, affective regulation, and the monitoring of internal states. Elevated *β* activity is typically correlated with vigilance, cognitive engagement, and tense arousal. Consequently, the suppression of *β* activity at FZ suggests a rapid mitigation of frontal midline tension. This phenomenon is likely attributable to the relaxation response induced by the respiratory protocol. By stabilizing the respiratory rhythm, the intervention redirects the individual’s attention from external stimuli and affective reactivity toward the physiological act of breathing, thereby lowering cognitive tension and hyperarousal. This interpretation is directionally consistent with the subjective POMS findings, which showed reductions in Tension, Confusion, and TMD scores. Because no direct correlation analysis was conducted between POMS changes and EEG changes, these subjective and neurophysiological results are best regarded as complementary and mutually supportive findings. These findings align with the systematic review by (5), who postulated that slow breathing techniques promote affective control and mental well-being by regulating autonomic and central nervous system activities, alongside enhancing psychological flexibility. The corresponding physiological *β* reduction and psychological symptom alleviation in this study validate that real-time respiratory modulation actively improves affective states by mitigating tense arousal.

### 4.2 Midline beta redistribution

This study found no significant differences in the F3/F4 Frontal Asymmetry Index (AI) for either the *β* or *θ* bands, indicating that real-time respiratory modulation does not elicit stable lateralized shifts in prefrontal activity. This is presumably because the protocol emphasizes respiratory awareness, rhythmic control, and somatic relaxation, rather than directly provoking distinct approach or avoidance motivations typically associated with hemispheric asymmetry. In contrast, the FZ/FCZ Midline Differential Index (MDI) for the *β* band decreased significantly post-intervention, indicating a relative reduction in *β* power at FZ compared to FCZ. This reveals that the neurophysiological impact of respiratory modulation is primarily characterized by the modulation of relative *β* distribution between the frontal midline and central regions, driving a spatial redistribution of activity along the anteroposterior midline, rather than lateralized changes. Given *β*’s association with vigilance and tense arousal, the lowered *β* MDI further corroborates that frontal midline tension decreases, allowing the individual to enter a more relaxed and stabilized regulatory state. This conclusion is consistent with research by (68), which demonstrated that controlled respiratory training modulates autonomic activity, diminishes negative affect, and specifically influences spatial EEG distributions, highlighting the role of midline cortical structures in respiratory-driven affective improvement.

### 4.3 Non-significant theta spatial tendency

Topographic difference maps showed a descriptive, non-significant spatial tendency of *θ* oscillations, with relative prefrontal enhancement and occipital reduction following the intervention. Because these *θ*-band changes did not reach statistical significance, they should be interpreted cautiously as trend-level observations rather than definitive *θ*-band effects. This spatial reconfiguration likely reflects the redistribution of attentional resources dictated by the task. Since the protocol requires sustained focus on breathing rhythms and internal somatic sensations, the prefrontal *θ* enhancement is indicative of increased internal attention, heightened respiratory awareness, and reinforced self-regulation. Conversely, the reduction in occipital *θ* suggests a decreased reliance on external visual information processing or low-frequency posterior cortical activity, pointing to a shift toward an internally directed regulatory state. This observation aligns with the findings of (16), whose randomized controlled trial established that short-term deep breathing directly influences EEG power and topographic distribution. In the current study, these *θ*-band spatial shifts did not achieve statistical significance at the single-channel level. Consequently, they must be interpreted conservatively as emerging spatial trends rather than definitive localized changes. Nevertheless, these patterns strongly correspond with the hypothesized mechanistic trajectory of respiratory induced neural redistribution.

### 4.4 Subjective affective improvement

Subjective assessments provided compelling behavioral evidence for the intervention’s efficacy, demonstrating a significant alleviation of negative affect and a concurrent enhancement of self-esteem. Post-intervention, participants experienced marked improvements in their overall affective states, evidenced by significant reductions in Tension, Anger, Fatigue, Depression, Confusion, and the comprehensive TMD score. The underlying mechanism driving this rapid improvement likely stems from the active deceleration and deepening of the respiratory rhythm, combined with directed attentional focus on the breath. This process effectively disrupts hyperarousal, bolsters the psychosomatic relaxation response, and transitions the individual from a state of psychological turbulence and confusion to one of stability. The concomitant decrease in negative affect and increase in self-esteem were directionally consistent with the reduction in FZ beta activity, suggesting that the subjective and EEG findings may provide complementary evidence of affective regulation. Furthermore, these results are highly consistent with the findings of (67), who demonstrated that diaphragmatic breathing not only mitigates negative affect and improves sustained attention but also blunts stress-related cortisol responses. In summary, real-time respiratory modulation acts as a potent psychomotor intervention that significantly enhances subjective affective experiences while concurrently stabilizing the underlying neural phenotypes associated with emotional distress.

## 5 Conclusion and future directions

### 5.1 Conclusion

This exploratory within-subject study provides preliminary evidence that the mechanism-driven, realtime respiratory modulation protocol may be associated with reductions in negative affective states and changes in cortical activity. At the behavioral level, post-intervention assessments utilizing the POMS questionnaire revealed significant reductions in Tension, Anger, Fatigue, Depression, Confusion, and Total Mood Disturbance (TMD) scores, accompanied by a marked increase in self-esteem. These findings suggest that the short-duration respiratory intervention may serve as a potential non-pharmacological strategy for rapidly alleviating negative psychological symptoms and elevating the overall affective state.

At the neurocognitive level, the intervention was associated with significant *β*-band modulation, whereas *θ*-band topographic changes were observed only as descriptive, non-significant spatial tendencies. Notably, *β*-band activity exhibited a pronounced downward trend in the FZ region, while the *θ* band showed only a descriptive, non-significant spatial tendency toward prefrontal enhancement and occipital reduction. Furthermore, while the intervention did not elicit significant lateralized shifts in the prefrontal cortex (as evidenced by the stable F3/F4 Frontal Asymmetry Index), it significantly altered the anteroposterior spatial distribution along the midline. The marked decrease in the *β*-band Midline Differential Index (MDI) indicates a relative reduction of *β* power at FZ compared to FCZ. Given the established correlation between *β* oscillations and vigilance, cognitive load, and tense arousal, this relative midline *β* suppression may reflect a rapid attenuation of frontal hyperarousal. Collectively, the integration of subjective and neurophysiological evidence demonstrates that real-time respiratory modulation regulates cortical networks associated with affective control and internal attention by reducing tense arousal, enhancing somatic awareness, and facilitating psychosomatic relaxation.

Crucially, these findings bridge the conceptual gap introduced earlier regarding the utility of respiration as a distinct psychomotor marker in computational psychiatry. By successfully capturing prefrontal EEG oscillatory dynamics, specifically frontal midline *β* suppression, this study identifies these neural signatures as potential computational phenotypes for evaluating affective regulation. Addressing the inherent limitations of traditional, long-term breathing regimens, such as low compliance and delayed efficacy, the proposed real-time framework offers a highly operational and rapid-onset solution tailored for acute affective disturbances. Ultimately, this research not only provides preliminary empirical evidence for respiration-based affective interventions but also accelerates the clinical translation of these mechanisms into digital therapeutics. It offers critical insights for optimizing real-time biofeedback parameters and designing adaptive, precision-driven tools for psychiatric evaluation and affective management.

### 5.2 Limitations

Despite the promising findings, several limitations of the current study must be acknowledged. First, the relatively modest sample size (N = 24 for the final analysis) may restrict the external validity and generalizability of the results. Second, the present study did not include a resting control group or a sham-breathing control condition, which limits the ability to fully distinguish the specific effects of the respiratory modulation protocol from non-specific factors such as time effects, expectancy effects, or general relaxation effects. Third, the current protocol did not systematically compare the differential neurophysiological modulatory effects of various respiratory patterns (e.g., diaphragmatic versus thoracic breathing). Fourth, the study relied primarily on EEG and subjective scales, omitting the synchronous acquisition of complementary physiological markers, such as heart rate variability (HRV) or cortisol levels, which could provide a more holistic view of autonomic and endocrine responses. Finally, the lack of strict control over potential moderating variables, such as baseline affective states and specific personality traits, may influence individual responsiveness to the real-time respiratory intervention.

### 5.3 Future directions

To address these limitations and further validate the computational phenotypes identified, future research should expand upon the current findings across multiple dimensions. Methodologically, multicenter trials with larger cohorts (*n* ≥ 100) are warranted to ensure demographic diversity and accommodate clinical heterogeneity. Such studies should explicitly include populations diagnosed with clinical affective disturbances, such as anxiety and depressive disorders. Furthermore, randomized crossover designs should be implemented to systematically evaluate and isolate the neural mechanisms underlying different respiratory patterns.

Regarding assessment methodologies, integrating long-term follow-up tracking with multimodal physiological monitoring will establish a more comprehensive evaluative framework. To further elucidate the neurobiological mechanisms, the application of concurrent multimodal neuroimaging techniques (e.g., simultaneous EEG-fNIRS) is highly recommended to decode the complex prefrontal-amygdala circuitry governing respiratory modulation. Finally, from a translational perspective, future efforts should prioritize the development of intelligent, biofeedback-driven real-time respiratory modulation systems and explore combinatorial therapeutic protocols. These advancements will systematically propel the scientific application of respiratory interventions in digital psychiatry, providing robust evidence-based support for mechanism elucidation, protocol optimization, and clinical translation.

## Data Availability

The raw data supporting the conclusions of this article will be made available by the authors, without undue reservation.
